# Supplementary material for: Multi‐dataset identification of innovative feature genes and molecular mechanisms in keratoconus
Source: J Cell Mol Med. 2024 Sep 19;28(18):e70079. doi: 10.1111/jcmm.70079 (PMC11412914; doi:10.1111/jcmm.70079)
Supplement: Supplementary file 2 — Material S2. [file JCMM-28-e70079-s002.zip › 7.GSEA analysis/6.SNORA74B/KEGG_PRIMARY_IMMUNODEFICIENCY.html]

Details for gene set KEGG\_PRIMARY\_IMMUNODEFICIENCY[GSEA]

|  || Dataset | SNORA74B.group.cls#h\_versus\_l |
| Phenotype | group.cls#h\_versus\_l |
| Upregulated in class | h |
| GeneSet | KEGG\_PRIMARY\_IMMUNODEFICIENCY |
| Enrichment Score (ES) | 0.18825153 |
| Normalized Enrichment Score (NES) | 0.5120015 |
| Nominal p-value | 1.0 |
| FDR q-value | 1.0 |
| FWER p-Value | 1.0 |
Table: GSEA Results Summary

  

Fig 1: Enrichment plot: KEGG\_PRIMARY\_IMMUNODEFICIENCY      
 Profile of the Running ES Score & Positions of GeneSet Members on the Rank Ordered List

  

| SYMBOL | TITLE | RANK IN GENE LIST | RANK METRIC SCORE | RUNNING ES | CORE ENRICHMENT || 1 | CD19 | na | 340 | 0.668 | 0.1502 | Yes |
| 2 | TNFRSF13C | na | 3685 | 0.271 | 0.1187 | Yes |
| 3 | IL7R | na | 3715 | 0.270 | 0.1826 | Yes |
| 4 | JAK3 | na | 5304 | 0.215 | 0.1883 | Yes |
| 5 | CD4 | na | 8358 | 0.128 | 0.1309 | No |
| 6 | BLNK | na | 9102 | 0.111 | 0.1361 | No |
| 7 | CD3D | na | 9511 | 0.102 | 0.1489 | No |
| 8 | TNFRSF13B | na | 9844 | 0.096 | 0.1622 | No |
| 9 | CD79A | na | 11215 | 0.071 | 0.1397 | No |
| 10 | CIITA | na | 13002 | 0.047 | 0.0995 | No |
| 11 | DCLRE1C | na | 13151 | 0.045 | 0.1061 | No |
| 12 | UNG | na | 13329 | 0.044 | 0.1114 | No |
| 13 | AICDA | na | 14603 | 0.029 | 0.0816 | No |
| 14 | IGLL1 | na | 16340 | 0.011 | 0.0343 | No |
| 15 | RFXANK | na | 16566 | 0.009 | 0.0299 | No |
| 16 | CD3E | na | 16594 | 0.008 | 0.0311 | No |
| 17 | ICOS | na | 17341 | 0.001 | 0.0099 | No |
| 18 | PTPRC | na | 18235 | -0.009 | -0.0138 | No |
| 19 | BTK | na | 18390 | -0.011 | -0.0157 | No |
| 20 | TAP2 | na | 18623 | -0.013 | -0.0192 | No |
| 21 | IKBKG | na | 20820 | -0.038 | -0.0734 | No |
| 22 | AIRE | na | 21715 | -0.048 | -0.0877 | No |
| 23 | CD8B | na | 21807 | -0.049 | -0.0785 | No |
| 24 | ADA | na | 22368 | -0.056 | -0.0811 | No |
| 25 | TAP1 | na | 22682 | -0.061 | -0.0756 | No |
| 26 | RFX5 | na | 23524 | -0.073 | -0.0823 | No |
| 27 | RFXAP | na | 25266 | -0.103 | -0.1079 | No |
| 28 | RAG1 | na | 25669 | -0.110 | -0.0930 | No |
| 29 | IL2RG | na | 26016 | -0.117 | -0.0749 | No |
| 30 | CD8A | na | 26376 | -0.125 | -0.0554 | No |
| 31 | LCK | na | 28532 | -0.180 | -0.0744 | No |
| 32 | ZAP70 | na | 28920 | -0.193 | -0.0394 | No |
| 33 | CD40LG | na | 30278 | -0.236 | -0.0221 | No |
| 34 | RAG2 | na | 31206 | -0.278 | 0.0179 | No |
| 35 | CD40 | na | 32420 | -0.349 | 0.0665 | No |
Table: GSEA details [plain text format]

  

Fig 2: KEGG\_PRIMARY\_IMMUNODEFICIENCY      
 Blue-Pink O' Gram in the Space of the Analyzed GeneSet

  

Fig 3: KEGG\_PRIMARY\_IMMUNODEFICIENCY: Random ES distribution      
 Gene set null distribution of ES for **KEGG\_PRIMARY\_IMMUNODEFICIENCY**
